# Ovarian Tumor Removed, per Vias Naturales, by Catheterism of the Fallopian Tubes

**Published:** 1851-09

**Authors:** 


### ECLECTIC DEPARTMENT,

### AND SPIRIT OF THE MEDICAL PERIODICAL PRESS.

*Ovarian Tumor Removed, per vias Naturales, by Catheterism of the Fallopian Tubes.* Reported by SAMUEL A. CARTWRIGHT, M. D., New Orleans.

March 10th, 1850, I sent for Dr. Warren Stone, to consult him in regard to the propriety of extirpating a very large and hard ovarian tumor in a patient of mine, Mrs. * * *, a small, delicately-formed lady, of sanguine temperament and scrofulous constitution, lately from the country. The patient herself wanted an operation performed, and came to the city for that purpose. When I told her that it would require an incision two feet long to extirpate so large a tumor, she replied, that she did not care if it were three feet, as she had rather die than live to suffer as she did. A tormenting strangury, from the pressure of the tumor on the bladder, annoyed her very much day and night. She was about nineteen years of age, had been married two years, and was very feeble, pale and emaciated. She said that the tumor had been growing from her earliest recollection, but it had not become so large as to incommode her much, until after her marriage; she had taken iodine and its preparations for a long time; had been twice salivated, and so far from deriving any benefit, grew weaker, and the tumor continued to enlarge. She was also afflicted with bronchitis and ulceration of the throat, which she attributed to salivation. The tumor made her look as large as a woman in the ninth month of pregnancy; it was hard and irregular to the touch, and seemed to arise from the left ovarium; it would incline from side to side with the position of the body; a prolongation of the tumor had slipped down between the bladder and uterus, and so much compressed the vagina as to be in the way of a speculum examination. As the case was beyond the reach of medicine, the resources of surgery were invoked. After a careful examination, Dr. Stone came to the conclusion that a surgical operation would be too hazardous, and in all probability fatal, in consequence of adhesion of the tumor to the bladder and contiguous viscera.

The patient was put on a course of proto-iodide of mercury, combined with cicuta; the tincture of pareira brava root advised for the relief of the irritation of the bladder. This treatment was continued for seven or eight days, the patient growing weaker and the tumor larger. The disease of the throat became so annoying that I found it necessary to apply the nitrate of silver frequently to the ulcerated and inflamed tonsils, and to substitute tonics for the iodide of mercury and cicuta.

On the 18th of March, the patient consented to a speculum examination. The uterus was rather under the usual size; there was no leucorrhœal discharge, congestion or inflammation; the mucous surfaces were in a state of anæmia, being pale and exsanguious. A very small gum elastic catheter, with a wire in it, after repeated efforts, was introduced into the uterine cavity. The passage of the catheter through the coarctation, called the os internum, gave some pain, and caused a faintish, sick sensation; but this is nearly always the case in probing a healthy uterus, and the operation requires some address and a proper instrument, or it cannot be effected. The small catheter was withdrawn, and a larger-sized instrument was passed with some difficulty through the cavity of the cervix into the uterus; it penetrated about two inches; on being withdrawn, a little blood, as usual, followed. I now concluded to try catheterism of the left Fallopian tube. With this view, the catheter, containing a wire, was flexed like the male catheter and passed through the fusiform cavity of the neck into the triangular cavity of the uterus itself; the wire was withdrawn about half an inch, so as to make the point of the instrument more flexible, and was carried forward in the direction of the ostium uterinum of the left Fallopian tube. It entered the tube after a few trials, and after penetrating about an inch, it seemed to enter a cavity or expansion of the tube itself; it was pushed forward about an inch and a half more, seeming in its passage to encounter a soft, yielding substance; it was then withdrawn; a glutinous substance followed its withdrawal, which I recognized to be a hydatid formation. The same catheter, with a very tapering point, was dipped in a solution of nitrate of silver, a drachm to an ounce. Several minims of the solution were drawn into it by working the wire in the calibre of the instrument; it was then passed through the uterine cavity into the Fallopian tube, until it had penetrated the tube three inches, when it was moved about in the cavity of the tube, and the wire moved so as to eject the caustic solution through the eyes of the catheter, among the hydatid cysts that the instrument had reached; on withdrawing it, a semi-membranous, tenacious substance, with dark specks interspersed through it, not unlike frog-spawn, presented itself at the mouth of the uterus, seeking an exit, but too thick and glutinous to pass freely. Finding it too soft and yielding to be drawn away with the forceps, a little raw cotton was passed around a probe, so as to entangle the viscid substance; and by turning the probe the stringy matter was wound around it, and pulled out of the uterus in long mucilaginous ropes. The supply seemed to be inexhaustible. The patient being much fatigued, the operation of drawing away the hydatids was at length suspended; nevertheless they continued to come away, *per vias naturales*, for a week or more. In the mean time, the tumor was reduced to less than half its former size, and grew softer and less painful. The catheterism of the Fallopian tube, with the catheter filled with a strong solution of nitrate of silver, was again repeated on the 3d of April, the 1st, 7th, and 13th of May. At the last operation no more hydatids or viscid fluid was brought away; but at all the other operations, they were not only brought away at the time, but continued to pass off for a week or more after each catheterism.

The day after the last operation, the patient left town for the sea shore; her health had begun to improve rapidly, her pains were gone, and her abdomen was reduced to near its natural size. While absent, her health appeared to be entirely reinstated.

Last autumn she returned to the city quite well, though some fullness and hardness could still be felt in the hypogastric region, the effects of the former adhesions, verifying the accuracy of the diagnosis made by Dr. Stone, of whose skill in surgery New Orleans is justly proud. Soon after her return she had an attack of fever, which, as is usual, sought out the weakest part, and the ovarian region again became the seat of painful sensations, which, with the distension from dyspeptic flatulence, made her apprehensive, for some months, that she was not cured; but her general health improved in the course of the winter, and she was enabled to dance, waltz, and walk about town as actively as almost any other woman. Although she suffers somewhat from painful distension of the abdominal and pelvic regions during her menstrual periods, and is dyspeptic and flatulent at such times, yet, when that is over, her form is quite sylph-like. Her bronchial disease is cured, and the leucophlegmasia is giving way to the rosy hue proper to her original sanguine temperament.

As this is the first case of ovarian tumor, as far as I know, which has been treated by catheterism of the Fallopian tubes, I have thought proper to report it. I do not consider the operation as always a difficult one; because, when the ovaries are in a morbid state, the Fallopian tubes are, in general, much more easily catheterized than in the healthy condition. I doubt its practicability in a state of health; possibly it might be effected during the catamenial period. The same important practical law obtains in regard to the uterus itself, it being generally easier to probe when in a morbid state. I have succeeded in curing some cases of dysmenorrhœa and sterility by catheterism of the Fallopian tubes, selecting the proper time for the operation; but as its virtues in this respect are already known to the profession, it is unnecessary to dwell upon the subject farther than to say, that New Orleans can show some as unquestionable evidences of its efficacy in sterility as London.—*New Orleans Medical and Surgical Journal.*

